# Cortical Activation during Action Observation, Action Execution, and Interpersonal Synchrony in Adults: A functional Near-Infrared Spectroscopy (fNIRS) Study

**DOI:** 10.3389/fnhum.2017.00431

**Published:** 2017-09-05

**Authors:** Anjana N. Bhat, Michael D. Hoffman, Susanna L. Trost, McKenzie L. Culotta, Jeffrey Eilbott, Daisuke Tsuzuki, Kevin A. Pelphrey

**Affiliations:** ^1^Department of Physical Therapy, University of Delaware Newark, DE, United States; ^2^Department of Psychological and Brain Sciences, University of Delaware Newark, DE, United States; ^3^Biomechanics and Movement Science Program, University of Delaware Newark, DE, United States; ^4^The George Washington Autism Institute, George Washington University Washington, DC, United States; ^5^Department of Language Sciences, Tokyo Metropolitan University Tokyo, Japan

**Keywords:** interpersonal synchrony, imitation, autism, fNIRS, mirror neuron system, action observation, action execution

## Abstract

**Introduction:** Humans engage in Interpersonal Synchrony (IPS) as they synchronize their own actions with that of a social partner over time. When humans engage in imitation/IPS behaviors, multiple regions in the frontal, temporal, and parietal cortices are activated including the putative Mirror Neuron Systems (Iacoboni, 2005; Buxbaum et al., 2014). In the present study, we compared fNIRS-based cortical activation patterns across three conditions of action observation (“Watch” partner), action execution (“Do” on your own), and IPS (move “Together”).

**Methods:** Fifteen typically developing adults completed a reach and cleanup task with the right arm while cortical activation was examined using a 24-channel, Hitachi fNIRS system. Each adult completed 8 trials across three conditions (Watch, Do, and Together). For each fNIRS channel, we obtained oxy hemoglobin (HbO_2_) and deoxy hemoglobin (HHb) profiles. Spatial registration methods were applied to localize the cortical regions underneath each channel and to define six regions of interest (ROIs), right and left supero-anterior (SA or pre/post-central gyri), infero-posterior (IP or angular/supramarginal gyri), and infero-anterior (IA or superior/middle temporal gyri) regions.

**Results:** In terms of task-related differences, the majority of the ROIs were more active during Do and Together compared to Watch. Only the right/ipsilateral fronto-parietal and inferior parietal cortices had greater activation during Together compared to Do.

**Conclusions:** The similarities in cortical activation between action execution and IPS suggest that neural control of IPS is more similar to its execution than observational aspects. To be clear, the more complex the actions performed, the more difficult the IPS behaviors. Secondly, IPS behaviors required slightly more right-sided activation (vs. execution/observation) suggesting that IPS is a higher-order process involving more bilateral activation compared to its sub-components. These findings provide a neuroimaging framework to study imitation and IPS impairments in special populations such as infants at risk for and children with ASD.

## Introduction

Interpersonal Synchrony (IPS) and imitation, both involve observation of other's actions as well as execution of observed actions. Imitation involves a series of discrete actions to reproduce a partner's actions, e.g., playing a Simon Says game (Meltzoff, 2007; Nadel, 2015). Imitation tasks typically include discrete dyadic actions such as tongue protrusions, eye blinks, or communicative gestures to a partner (Nadel, 2015) as well as triadic actions such as tool use (Smith and Bryson, 2007). When actions are similar, engaging in IPS is relatively complex as it involves continuous rhythmic actions with moment-to-moment synchronization over time, (e.g., marching in a band) compared to imitation that involves reproducing a motor pattern for a finite number of actions (e.g., reproducing a marching sequence, say, “March like this—1, 2, 3, 4”) (Marsh et al., 2009; Vicaria and Dickens, 2016). IPS between individuals has been studied across a variety of continuous rhythmic arm movements i.e., finger tapping on a surface (Nowicki et al., 2013; Rabinowitch and Knafo-Noam, 2015), reaching for objects (Schmitz et al., 2017), and swinging pendulums (Fitzpatrick et al., 2016), leg movements such as walking (Wiltermuth and Heath, 2009), as well as whole body movements such as swaying (Sofianidis et al., 2012), bouncing (Cirelli et al., 2016), and rocking (Marsh et al., 2009). IPS actions can be dyadic such as clap-tap hand gestures (Tunçgenç and Cohen, 2016) or triadic goal-directed actions such as reaching/placing of items, swinging pendulums, or drumming (Kleinspehn-Ammerlahn et al., 2011; Fitzpatrick et al., 2016; Schmitz et al., 2017). Taken together, there are clear commonalities and distinctions between imitation and IPS behaviors with imitation acts requiring correspondence during discrete actions and IPS behaviors demanding sustained synchrony over time.

Imitation and IPS allow humans to learn various skills such as social gestures, object-based gestures and tool-use, as well as adaptive and functional skills (Dewey, 1995; Carpenter et al., 1998; Jones, 2007; Meltzoff, 2007). Young children imitate novel object-related actions of adult partners as well as intentional social gestures such as pointing as early as 18 months of age (Meltzoff, 1988; Carpenter et al., 1998). Older children learn various adaptive functional skills such as tying shoelaces or buttoning shirts through observation of caregivers and peers (Dewey, 1995). Furthermore, IPS between partners such as during drumming or walking facilitates social connections between partners leading to feelings of closeness, liking, and trust as well as prosocial behaviors of helpfulness/cooperation (Wiltermuth and Heath, 2009; Kirschner and Tomasello, 2010; Vicaria and Dickens, 2016). Both, imitation and IPS abilities are significantly impaired in neurological populations such as individuals with Autism Spectrum Disorder (ASD) (Rogers et al., 2003; Marsh et al., 2009; Fitzpatrick et al., 2016). Hence, the present study aims to understand the neural mechanisms underlying IPS performance in healthy adults during naturalistic social interactions using a fundamental reaching task.

Imitation and IPS share similar basic perceptuo-motor and cognitive processes; hence, the underlying neural substrates should also be similar (Vicaria and Dickens, 2016). The neural substrates for IPS have not been studied using traditional functional Magnetic Resonance Imaging (fMRI) techniques; however, fMRI studies provide substantial evidence for widespread cortical activation including the frontal, parietal, and occipito-temporal regions not only during imitation but also during other motor, language, and social functions (Iacoboni, 2005; Iacoboni and Dapretto, 2006; Caspers et al., 2010); hence, it would be reasonable to postulate that these regions may be activated during IPS behaviors as well. Next, we describe evidence from studies on imitation control from two different neuroscientific frameworks—the putative Mirror Neuron Systems (MNS, Iacoboni, 2005; Cattaneo and Rizzolatti, 2009) and the neurocognitive models of gestural control/Apraxia (Caspers et al., 2010; Buxbaum et al., 2014). In terms of regional activation, multiple cortical regions are activated during imitation performance; however, certain cortical regions are said to be consistently active during action observation, action execution, and imitation; forming an important imitation network (Iacoboni, 2005; Cattaneo and Rizzolatti, 2009): (i) the Inferior Parietal Lobule (IPL), the intraparietal sulcus of the parietal lobe, the Supramarginal Gyrus (SMG) and the Angular Gyrus (AG), (ii) the Superior Temporal Sulcus (STS) regions of the temporal lobe including the Superior and Middle Temporal Gyri (STG and MTG), and (iii) the Inferior Frontal Gyrus (IFG) and ventral Premotor Cortex (vPMC) of the frontal lobe. The imitation network does not function independently and their sub-regions continuously interact with each other and other brain regions depending on context/nature of the imitation/IPS tasks (Gazzola and Keysers, 2009; Iacoboni, 2009; Turella et al., 2009; Jack et al., 2011; Vrticka et al., 2013). Additional brain regions activated during imitation behaviors may include other visual, social, and motor regions important for visual/social perception, working memory, motor planning, and action execution including dorsolateral prefrontal cortices, premotor cortices, primary and supplementary/pre-supplementary motor cortices, cingulate/insular cortices, cuneus/precuneus as well as subcortical structures such as the cerebellum and putamen (Gazzola and Keysers, 2009; Iacoboni, 2009; Turella et al., 2009; Jack et al., 2011; Vrticka et al., 2013). The imitation network formed by IPL, STS, IFG along with its connections with other regions are often discussed as part of the putative Mirror Neuron System and are suggested to play an important role in imitation and possibly IPS behaviors in humans (Iacoboni, 2005; Vicaria and Dickens, 2016).

The STS is reportedly more active during observation of biological motions vs. non-biological motion controls (Pelphrey et al., 2003). During imitation of a hammering task, it was found to be more bilaterally active than during pure observation or execution of the same task suggesting that it played a greater role beyond passive registration of biological motion, perhaps representing visuomotor correspondence between one's own action and that of the partner (Iacoboni and Dapretto, 2006; Molenberghs et al., 2010). The literature on gesture control and apraxia also suggests that the middle temporal gyri (lower portion of the STS) plays an important role in semantic action knowledge given its activation during action recognition in presence of pictures or word stimuli (Vingerhoets et al., 2009; Watson et al., 2013). Hence, it would be reasonable to detect some level of STS activation during observation of IPS behaviors.

Both the IFG and IPL regions of the putative MNS are said to be more active during observation and imitation of goal-directed, object-based actions vs. dyadic actions without objects (Iacoboni, 2005; Pokorny et al., 2015). Specifically, IPL may contribute to the motoric aspects of the imitated goal-directed action and IFG is often linked to encoding the goals of the action (Iacoboni, 2005, 2009). Within the gesture control/apaxia literature, the left IPL region is said to encode the kinematic aspects of gestures and is more activated when performing gestures sequences involving objects vs. non-prehensile actions (Buxbaum et al., 2006). Additionally, patients with left IPL damage had more impairments performing meaningless gestures compared to meaningful gestures; which was interpreted as a deficit in planning the kinematics of the gesture to be performed (Goldenberg and Hagmann, 1997). In the apraxia literature, IFG is said to be important for tool use and especially, the postural aspects of gesture production (Buxbaum et al., 2014). The meta-analysis on gestural control by Caspers et al. (2010) found both IFG and MFG to be active during gesture imitation tasks. Moreover, patients with left MFG and IFG stroke present with significant deficits in gesture imitation (Haaland et al., 2000; Goldenberg et al., 2007). Hence, both the putative MNS literature and the apraxia literature recognize the roles of IPL and IFG in planning of object/tool-based gestures including encoding of motor plans and action goals.

In terms of task-related differences in activation within the imitation network, the literature is more ambiguous when comparing action observation, execution, and imitation (Aziz-Zadeh et al., 2006; Montgomery et al., 2007; Molenberghs et al., 2010; Mengotti et al., 2012; Gatti et al., 2017). The first direct evidence for mirror neurons was observed in the premotor and parietal cells of the macaque brain (Rizzolatti and Craighero, 2004). They were found to be active when the animal performed a goal-directed action such as reaching and grasping for food and when they saw others doing the same actions. The initial human studies using fMRI also reported mirroring with similar levels of activation in the putative MNS regions during observation of other's actions, self-produced actions, and action imitation (Cattaneo and Rizzolatti, 2009). However, recent studies have reported different patterns of activation within the imitation network across action observation, action execution, and imitation tasks (Aziz-Zadeh et al., 2006; Montgomery et al., 2007; Molenberghs et al., 2010; Gatti et al., 2017). The first pattern involved greatest activation in the putative MNS regions during action imitation followed by action execution and lowest activation during action observation (Aziz-Zadeh et al., 2006). The second pattern involved similar levels of activation during action execution and imitation tasks involving object-related, goal-directed actions (Montgomery et al., 2007; Molenberghs et al., 2010). A third pattern involved greater activation during imitation compared to action execution and observation (Montgomery et al., 2007; Molenberghs et al., 2010; Gatti et al., 2017; Hamzei et al., 2017). In fact, each study reports mixed patterns of activation depending on the region of interest. Two studies reported some level of activation within the different putative MNS and related areas across different object-related actions with the parietal and premotor cortices being equally active in the imitation and execution conditions (left > right) and bilateral STS being more active during imitation compared to execution and observation (Montgomery et al., 2007; Molenberghs et al., 2010). Gatti et al. (2017) reported that object-related imitation led to greater activation in the right precentral gyrus, right IFG as well as bilateral STS compared to the observation and execution conditions. Overall, there appears to be a lack of consensus on how activations in the imitation networks differ across components of imitation. Our review of the current apraxia literature on gesture control provided converging evidence for imitation control. The regions considered important for gesture recognition, execution, and imitation include the three aforementioned regions of the imitation network, IPL, IFG, and STS (Buxbaum et al., 2005, 2014). A meta-analyses of multiple studies on gestural imitation reported activation in a large bilateral cortical network involving inferior parietal lobes, temporo-occipital, premotor, and primary somatosensory cortices (Caspers et al., 2010).

In terms of hemispheric differences, language is clearly left lateralized, sensori-motor control is contralateral in nature, whereas imitation control is said to be bilateral in nature (Aziz-Zadeh et al., 2006; Filimon et al., 2007; Caspers et al., 2010; Macuga and Frey, 2012). Aziz-Zadeh et al. (2006) asked healthy adults to either observe, execute or imitate static/dynamic hand tapping motions that were lateralized in terms of visual stimuli and hand use; only imitation motions led to greater bilateral activation in the IFG and IPL whereas activation was more contralateral in nature when the observation or execution condition stimuli/actions were lateralized to the right or left visual field/arm. Another study reported greater bilateral STS activation during gestural imitation (vs. observation and execution) whereas IPL activation was greater on the left side compared to the right, during both gestural imitation and execution (Montgomery et al., 2007). Some studies suggest that gestural imitation may be task-specific with imitation of bilateral actions leading to bilateral activation whereas imitation of unilateral actions leading to greater contralateral activation (Filimon et al., 2007; Macuga and Frey, 2012). However, a more comprehensive and quantitative meta-analysis confirms the initial hypotheses by Iacoboni and colleagues that activation associated with action imitation in frontal, parietal, and occipito-temporal regions are more bilateral in nature in spite of the tasks being unilateral (Caspers et al., 2010). Taken together, there is converging evidence from the MNS and gesture control/apraxia literature for the role of STS, IPL, IFG, and other sensori-motor regions during various components of imitation and we will further explore these regions as possible substrates for object-related IPS behaviors and its components, specifically, observation, execution, and IPS during repetitive reach-grasp actions.

fMRI has been a gold standard in neuroimaging research as it provides the most accurate measure of functional activation in the whole brain. However, it cannot be used during naturalistic social interactions and free limb movements due to its inability to handle motion artifacts. Participants have to remain absolutely still during fMRI procedures, slightest body movement can contribute to data errors/exclusion (Uddin et al., 2010). In contrast, fNIRS is a novel, non-invasive optical neuroimaging tool that provides robust data in the presence of movement artifacts. fNIRS has been used in various studies involving walking (Leff et al., 2011), dancing (Tachibana et al., 2011), as well as free arm movements (Egetemeir et al., 2011; Koehler et al., 2012). Sophisticated mathematical methods have been developed to address motion artifacts within pediatric fNIRS data (Hu et al., 2015). Furthermore, fMRI environments are unnatural due to the use of bright lights, loud noises, and constrained spaces. Participants have to perform tasks in a reclined body position within a narrow bore as they observe videos or perform actions limited to wrist and hand (Koehler et al., 2012). All of the imitation tasks described in the aforementioned putative MNS and apraxia literature were limited to hand gestures and video-based imitation as opposed to the everyday naturalistic imitation or IPS tasks with social partners. In contrast, fNIRS allows measurements during relatively naturalistic social interactions and while performing free limb movements in upright body positions (Ayaz et al., 2013; Tuscan et al., 2013). A Magneto-encephalography (MEG) study reported greater motor cortex activation in response to observation of live stimuli compared to 2D stimuli (Jarvelainen et al., 2001). Reader and Holmes (2015) reported greater imitation accuracy when individuals were shown live stimuli as compared to 2D stimuli. Although, fMRI studies have offered great insights into neural mechanisms of imitation, there is clearly added value in exploring naturalistic imitation/IPS paradigms as they could impact the level of brain activation and accuracy of performance.

The use of fNIRS in neuroimaging research has grown significantly over the last decade (Lloyd-Fox et al., 2010; Scholkmann et al., 2014); nevertheless, the majority of the studies focus on language or perceptual tasks (Bortfeld et al., 2009; Aslin, 2012; Jasinska and Petitto, 2014). A handful of studies have compared activation in the imitation networks during action observation, action execution, and imitation/IPS tasks (Egetemeir et al., 2011; Bolling et al., 2013). Based on our review of the fNIRS literature, only two studies have reported on activation in the putative MNS regions following action observation (Shimada and Abe, 2009; Bolling et al., 2013), two studies examined social cooperation during videogaming tasks (Cui et al., 2012; Liu et al., 2015) and three studies focused on joint action or imitation behaviors (Egetemeir et al., 2011; Koehler et al., 2012; Kajiume et al., 2013). Bolling et al. (2013) reported greater activation in the IPL, specifically, SMG during observation of biological motions compared to control, non-biological point light displays. Social inclusion led to further increase in STS activity compared to the social exclusion condition. Two studies exploring videogaming between competitors showed greater IFG and STS activation during observation or execution of different versions of cooperation vs. competition tasks (Shimada and Abe, 2009; Cui et al., 2012). Egetemeir et al. (2011) reported greater IPL activation during joint action with a social partner compared to solo actions performed during naturalistic table setting motions. Moreover, a lone fNIRS study in children with ASD has reported reduced activation in the IFG compared to typically developing controls during action observation tasks (Kajiume et al., 2013). Overall, it is feasible to study fNIRS-based cortical activation during imitation/IPS tasks with differential activation in various cortical regions.

The current study extends previous fNIRS research to a novel but simple reach and cleanup task so that it can be easily applied across different age groups of toddlers, children, and adults. The primary aim of our study was to compare fNIRS-based cortical activation between action observation, execution, and IPS conditions during a naturalistic reach and cleanup task between two adult partners. We hypothesized that the action execution or Do and IPS or Together conditions will lead to greater contralateral activation in the IPL and IFG (or fronto-parietal cortices) i.e., greater left-sided activation as our task involved right-handed, unilateral reach-grasp motions. Furthermore, during the IPS condition, we hypothesized an increase in ipsilateral cortical activation (i.e., pattern of bilateral activation) based on current literature findings. In terms of regional differences, we hypothesized that superior temporal cortex activation would be highest during the Watch condition whereas all three regions in the imitation network (inferior frontal, inferior parietal and superior temporal) would be activated during the Do and Together conditions. In terms of task-related differences, we hypothesized that the Do and Together conditions will lead to greater activation in the imitation network compared to the Watch condition. Lastly, the Together condition will result in an increase in ipsilateral cortical activation (i.e., pattern of bilateral activation) in the imitation network compared to the Do and Watch conditions. Findings from this study will provide the foundation for future studies comparing imitation and IPS performance between young and older children with and without ASD.

## Methods

### Participants

Fifteen typically developing, healthy adults between the ages of 19 and 27 years (Average: 22.6 ± Standard Error (SE): 0.7; 8 males and 7 females) participated in the study. The second author, a 22-year-old male, was the tester for all visits except for two participants, who were tested by the third author, a 22-year-old female. 14 participants were strongly right-handed and one participant was weakly right-handed based on a standard handedness questionnaire (Coren, 1992). The activation patterns for the last participant were consistent with that of our group results; hence, we have included the participant's data. All had normal or corrected to normal vision. Individuals were recruited using various online postings in local listservs, fliers, and through word of mouth. Participants completed a screening interview, which excluded individuals with any known neurological or psychiatric diagnoses or medication use. They also completed the Vineland Adaptive Behavioral Scales (VABS) interview (Volkmar et al., 1987) to provide measures of socialization (average percentile score = 66.0 ± 19.0), communication (average percentile score = 63.5 ± 15.5), daily living skills (average percentile score = 71.9 ± 19.1) as well as overall adaptive functioning (average percentile score = 72.9 ± 19.0) indicating typical levels of subdomain and overall adaptive functioning. The University of Delaware Institutional Review Board (UD IRB) approved this study protocol. Procedures for this study were carried out in accordance with the recommendations of UD IRB. All participants gave their written informed consent in accordance with the Declaration of Helsinki (as of 2008), prior to participation.

### Experimental procedures

**Figure 1:**
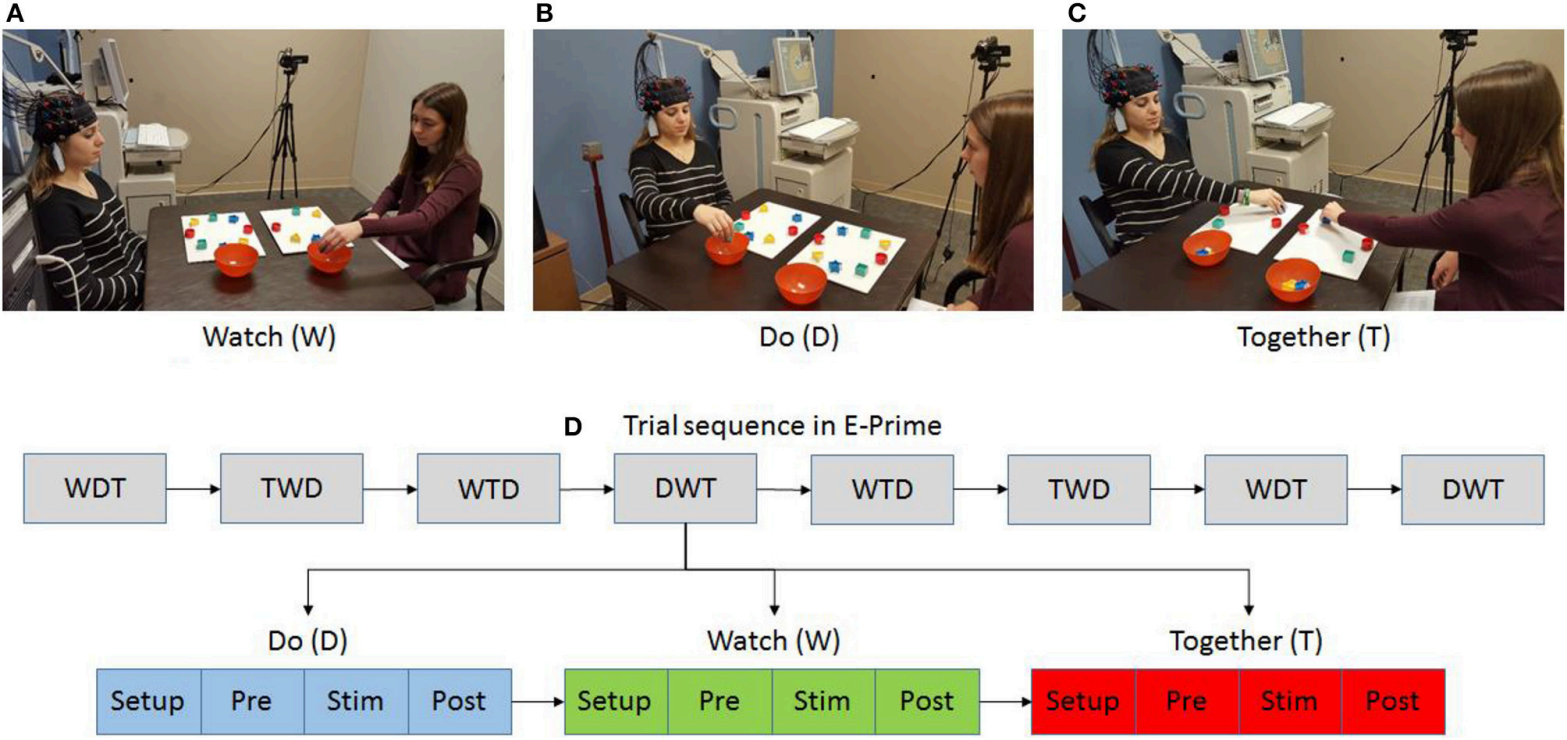
Experimental setup **(A–C)** and task sequence **(D)**. Written permission for publication of participant pictures has been taken.

**Figure 2:**
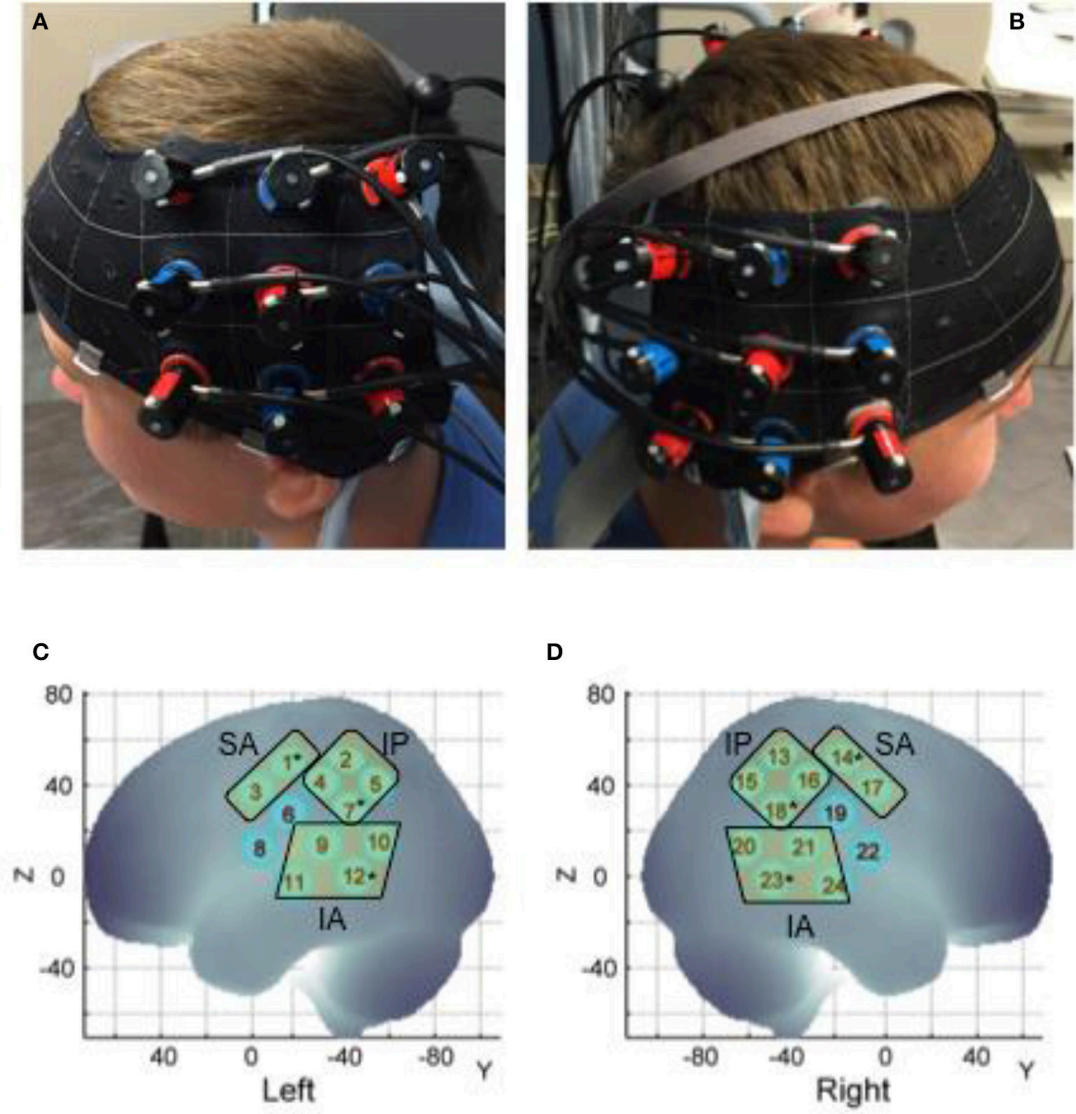
Probe placement **(A,B)** and spatial registration output **(C,D)**. Written permission for publication of participant pictures has been taken.

The participant and tester were seated at a table facing each other (Figures 1A,C). An fNIRS cap with two 3 × 3 probes was placed on the participant's head (Figures 2A,B). The task involved cleaning up an array of blocks into a container (Figures 1A,C). Participants completed three conditions using a pseudorandomized block design comprised of 8 blocks (i.e., a total of 24 trials). During the Watch (W) condition, the participant observed the tester pick up blocks in a sequential manner to complete the cleanup task. Participants were instructed to, “watch” the tester as they cleaned up the blocks. During the Do (D) condition, the participant cleaned up all blocks on their own using a sequence of their choice. Participants were instructed to “clean up the blocks on their own, in any order they like.” During the Together (T) condition, the tester led the cleanup of all blocks in a random order and the participant followed the tester continuously as he/she mirrored the cleanup activity by matching the block location/color and action components of pick, pass, and place. The tester always used the left hand and the participant used their right hand (Figure 1). Participants were instructed to “move together” and match their actions to the tester. The stimulation period ranged between 11 and 13 s (duration in seconds (s): W = 11.5 ± 1.6; D = 11.4 ± 1.1; T = 13.2 ± 1.5; duration for T condition was slightly greater than W and D due to task difficulty). We also included a pre-stimulation baseline (10 s) to account for any baseline drifts in the NIRS signal and a post-stimulation baseline (16 s) to allow the hemodynamic response to return to baseline before starting the next trial. Participants were asked to focus on a cross-hair on the front wall during both baseline periods.

### Data collection

Changes in oxygenation within each channel were captured using the Hitachi ETG-4000 system (Hitachi Medical Systems, Tokyo, Japan) (Sampling Rate: 10 Hz). Two 3 × 3 optode sets consisting of five infrared emitters and four receivers (i.e., 24 data channels) were positioned over bilateral fronto-parietal and temporal regions. In terms of vertical alignment, we aligned the middle column of each optode with the tragus mark of the ear below (Figure 2). The lowermost row of the optode set was aligned with the T3 position of the International 10–20 system (Klem et al., 1999; Jurczak et al., 2007), (Figure 2). An adjacent pair of probes, 3 cm apart, acted as an emitter and receiver for two wavelengths of infrared light (695 and 830 nm). The infrared light passes through the skull following a banana-shaped trajectory and reaches the cortical area approximately below the midpoint of any two probes. The change in infrared light attenuation can be used to calculate the changes in concentrations of oxygenated (HbO_2_) and deoxygenated hemoglobin (HHb) chromophores per channel using the Modified Beer-Lambert Law. We expect neural activation within a region to increase the concentration of HbO_2_ and decrease that of HHb (Lloyd-Fox et al., 2010). These data are exported within an output file in the comma-separated values (.csv) format and later post-processed. E-Prime presentation software (version 2.0) from a Windows PC triggered the Hitachi fNIRS system via a serial port to mark the baseline and stimulation periods, also stored in the csv output. The monitor relaying the onset and offset time of the clean up or set up period and condition type was seen by the tester and conveyed to the participant. The entire session was videotaped using a camcorder that was synchronized with the Hitachi fNIRS system.

### Spatial registration approach

At each session we recorded the 3D location of the standard cranial landmarks (nasion, inion, right and left ear) as well as 3D locations of each fNIRS probe w.r.t. a reference coordinate system using the ETG software and hardware. We applied the anchor-based, spatial registration method developed by Tsuzuki et al. (2012) to transform the 3D spatial location of each channel to the Montreal Neurological Institute (MNI)'s coordinate system for adult brains (see Figure 2 and Table 1). We used structural information from an anatomical database of 17 adults (Okamoto et al., 2004) to provide estimates of channel positions within a standardized 3D brain atlas (Tsuzuki et al., 2012). The estimated channel locations were anatomically labeled using the LONI Probabilistic Brain Atlas (LPBA) based on MRI scans of 40 healthy adults (Shattuck et al., 2008). For each channel location, we also estimated the spatial uncertainty due to inter-subject variability in holder placement (Mean SD = 11 mm approx., Range = 8–12 mm). We were interested in covering the putative MNS regions, namely, STS, IPL, and IFG and primary sensorimotor cortices (i.e., pre and post-central gyri). Based on the regions covered by our channels, we determined three regions of interest (ROIs) on each side (see Table 1): (i) the infero-anterior (IA) region included channels over the superior and middle temporal gyri (or superior temporal cortices or STS, see Figure 2B) and included left channels 9, 10, 11, and 12 and right channel 20, 21, 23, and 24, (ii) the infero-posterior (IP) region included channels over the inferior parietal gyri, the supramarginal and angular gyri (or the inferior/posterior parietal cortices or IPL, see Figure 2B) and included left channels 2, 4, 5, and 7 and right channels 13, 15, 16, and 17, and (iii) the supero-anterior (SA) region channels were over the precentral and post-central gyri and portions of IFG (or fronto-parietal cortices, Pre/Post-CG, see Figure 2B) and included left channels 1 and 3 and right channels 14 and 17. Unfortunately, our supero-anterior channels mostly covered pre/post-central gyri and somewhat covered the IFG and related areas due to probe size limitations. As is seen in Table 1, channels 6 and 8 on the left side and their right-sided homologues (channel 19 and 22) did not fall within the same ROIs. Data from these channels have been excluded to avoid spatial uncertainty within the averaged activation data. In this way, we were able to assign 20 out of the 24 channels to one of the aforementioned ROIs.

**Table 1:** Assignment of channels to regions based on anchor registration.

**Channel**	**X**	**Y**	**Z**	**Inferior frontal gyrus, opercular**	**Middle frontal gyrus**	**Rolandic operculum**	**Precentral gyrus**	**Postcentral gyrus**	**Inferior parietal lobule**	**Superior parietal gyrus**	**Supra marginal gyrus**	**Angular gyrus**	**Heschl gyrus**	**Superior temporal gyrus**	**Middle temporal gyrus**	**Inferior temporal gyrus**	**Middle occipital lobe**	**Assigned region**
**1**	−57.667	−16.667	52.667					0.730	0.183		0.087							SuperoAnterior
2	−57.000	−43.000	54.000					0.011	0.989									InferoPosterior
3	−61.000	−2.667	39.667				0.320	0.680										SuperoAnterior
4	−65.333	−31.333	43.667						0.235		0.765							InferoPosterior
5	−59.000	−56.667	42.667						0.533		0.077	0.391						InferoPosterior
6	−68.000	−17.333	29.333					0.601			0.399							Excluded^*^
**7**	−67.000	−44.667	30.667						0.006		0.884			0.109				InferoPosterior
8	−66.667	−3.667	13.667	0.026		0.255		0.548					0.026	0.145				Excluded^**^
9	−69.667	−32.333	15.333								0.223			0.539	0.238			InferoAnterior
10	−65.000	−58.333	16.333								0.081	0.081		0.155	0.684			InferoAnterior
11	−70.000	−19.333	−1.667											0.125	0.875			InferoAnterior
**12**	−69.333	−46.667	1.333											0.006	0.994			InferoAnterior
13	56.000	−47.000	55.667						0.882	0.114	0.004							InferoPosterior
**14**	59.000	−19.000	54.000		0.012		0.031	0.683	0.050		0.224							SuperoAnterior
15	57.000	−61.667	43.000						0.398			0.602						InferoPosterior
16	67.000	−34.333	44.333						0.116		0.884							InferoPosterior
17	64.000	−5.000	40.667				0.293	0.707										SuperoAnterior
**18**	66.667	−49.000	30.000						0.007		0.420	0.370		0.203				InferoPosterior
19	70.000	−21.333	29.333					0.080			0.917			0.003				Excluded^*^
20	62.667	−62.667	14.667									0.025		0.077	0.895		0.004	InferoAnterior
21	72.000	−36.333	14.667								0.003			0.844	0.153			InferoAnterior
22	69.667	−7.667	12.333			0.163		0.367					0.013	0.457				Excluded^**^
**23**	69.333	−50.667	−0.333												0.911	0.089		InferoAnterior
24	73.000	−22.667	−2.333											0.460	0.540			InferoAnterior

*Each channel is modeled as a sphere and the results from anchor registration provide how much fraction of the sphere belonged to each cortical gyrus. A single region was assigned to each channel based on dominance. For example, the majority of channel 2 was over IPL (99%); hence, it was assigned to the IP region. Color shading helps discern the ROI assigned to each channel based on coverage. Yellow depicts the SA region, cream depicts the IP region, and green depicts the IA region*.

*Channels 1–12 belong to the left side and channels 13–24 belong to the right side*.

* *Channels 6 and 19 were not assigned to any region as the anchor registration showed inconsistency in terms of the region assigned and were non-homologous. Channel 6 primarily belonged to the Post-central Gyrus, whereas its homologue on the right side (Channel 19) primarily belonged to the Supramarginal Gyrus*.

** *Similarly, Channels 8 and 22 were not assigned to any region as the anchor registration showed inconsistency in terms of the regions assigned and were ultimately, non-homologous. Channel 8 primarily belonged to the Post-central Gyrus, whereas its homologue on the right side (Channel 22) primarily belonged to the Superior Temporal Gyrus*.

*For channel-level analysis discussed in the Supplementary Material section, we chose channels 1 (L) and 14 (R) for the SA region (i.e., covering Pre/Post-CG), channels 12 (L) and 23 (R) for the IA region (i.e., covering STS including STG and MTG), and channels 7 (L) and 18 (R) for the IP region (i.e., over SMG and AG) based on which single channel had maximum coverage over a certain ROI (see bolded channel numbers in column 1 of this table)*.

### Data analysis

**Figure 3:**
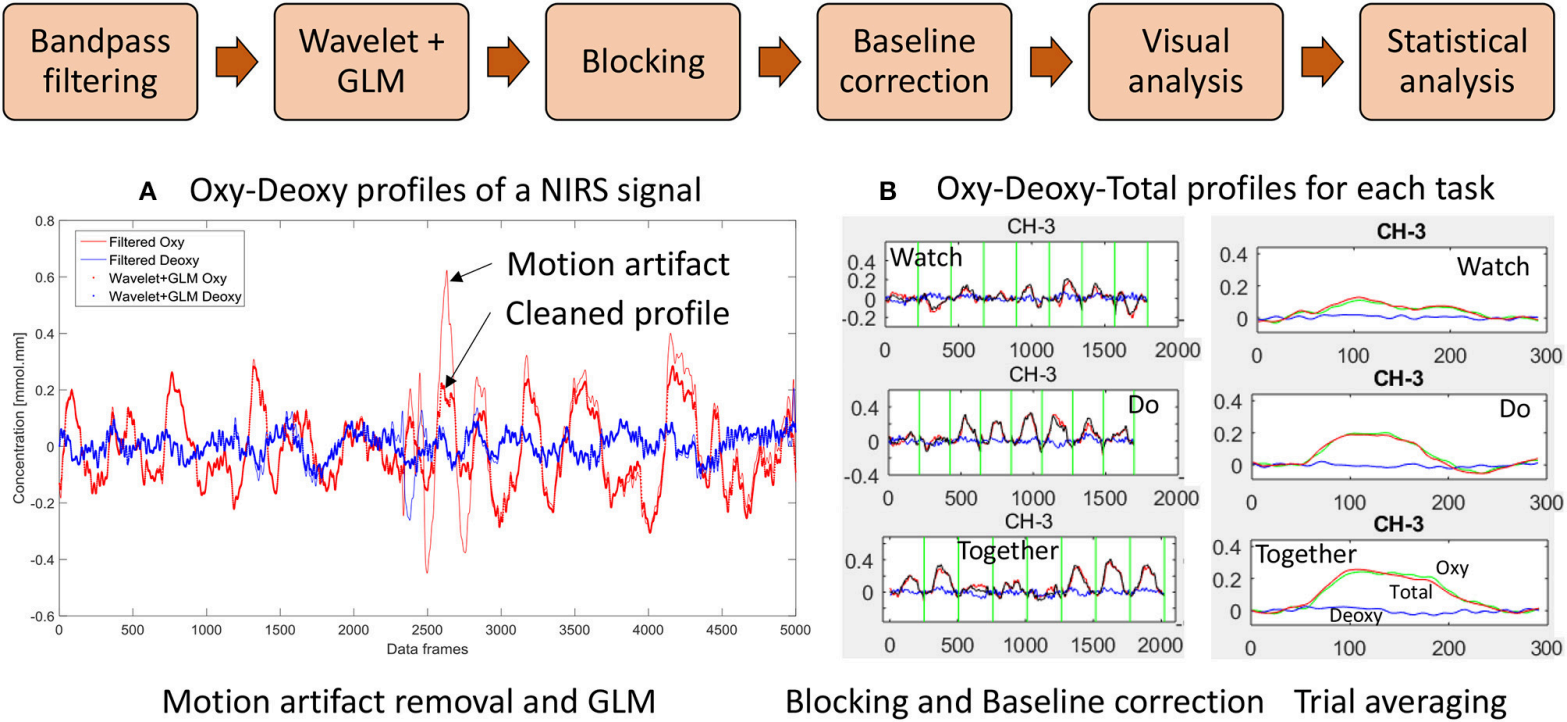
Data processing workflow: **(A)** Filter, wavelet and GLM of NIRS signal and **(B)** Trial-by-trial view and Average view of Oxy Hb (HbO_2_), Deoxy Hb (HHb), and Total Hb (HbT) profiles for a given channel. (W, D, T) from 5 s before to 24 s after start of stimulation. Data have been averaged across trials and participants.

**Figure 4:**
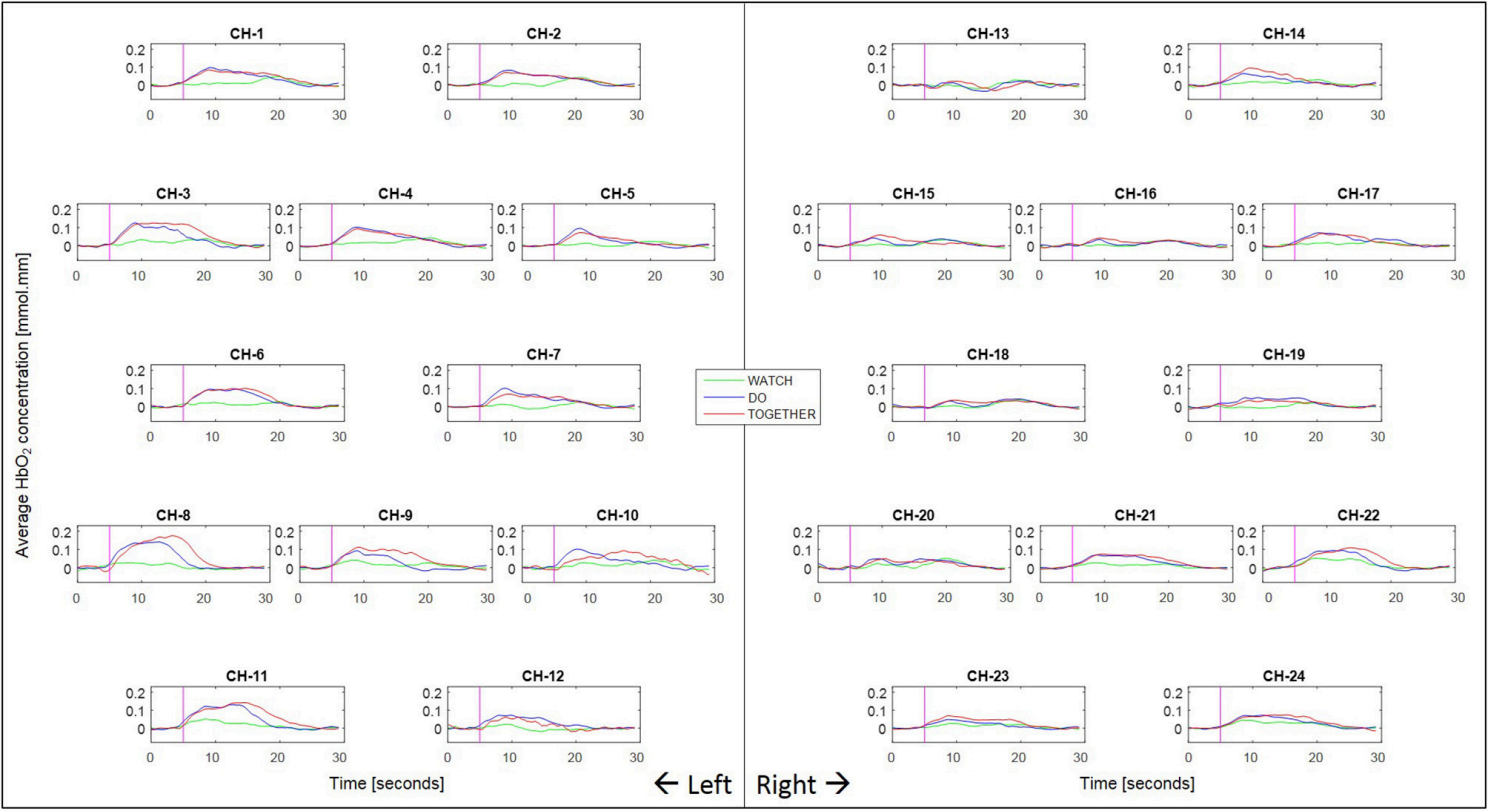
Second to second blocked HbO_2_ data per condition and channel. Pink vertical line denotes the start of the stimulation period and the following 240 frames across the stimulation (11–13 s) and post-baseline (13–11 s) period. The sampling frequency of the fNIRS system was 10 Hz (i.e., 10 data frames per second were collected).

We have incorporated functions from open-source software such as Hitachi POTATo (Sutoko et al., 2016) and Homer-2 (Huppert et al., 2009) within our own custom MATLAB (The Mathworks Inc., Natick, MA) codes to analyze the .csv output from ETG-4000 (see data processing steps in Figure 3). Data processing also included significant re-orgnization and pooling of data across trials and participants using our own custom APL (Dyalog Ltd.) codes. Signals from each channel were band-pass filtered between 0.01 and 0.5 Hz to remove lower or higher frequencies associated with body movements and other physiological signals such as respiration, heart rate, skin blood flow, etc. For motion artifact removal, we used the wavelet method as implemented in the Homer-2 software (Sato et al., 2006; Huppert et al., 2009); which is considered the most robust and effective method (Hu et al., 2015). General Linear Model (GLM—as implemented in the Homer-2 software) was used to estimate the hemodynamic response function using Gaussian basis functions and a third order polynomial drift regressor (Huppert et al., 2009). For baseline correction, the linear trend between the pre-trial baseline and the post-trial baseline was calculated and subtracted from values in the stimulation period as implemented within Hitachi POTATo (Sutoko et al., 2016). An average HbO_2_ and HHb value was obtained for the stimulation period of each trial. The range of HbO_2_ data were significantly greater than HHb data. Moreover, HbO_2_ profiles have a greater signal to noise ratio compared to HHb and therefore fNIRS literature most often reports HbO_2_ profiles (Sato et al., 2005). For visual representation, HbO_2_ and HHb profiles for each stimulation and post-baseline period were averaged across all trials for each condition and each channel for 24 s (13 s of stimulation and 11 s of post-baseline) after the start of each trial to show the second-to-second changes in each channel and condition, see Figure 4.

### Video data coding

We scored each session's video for percent erroneous trials in terms of IPS (a one-block or greater lag in synchrony with partner, 11.9 ± 12.9%), motor coordination (inaccurate grasping of block or bumping of container, 8.9 ± 9.4%), additional head or body movements (obvious head or trunk movements, 6.4 ± 9.1%), extraneous social interactions (speaking during trials, 0.5 ±1.5%) and/or probe displacement errors (probe displaced from holder, 0.6 ± 1.5). We established ~98% intra-rater and >85% inter-rater reliability for the aforementioned error codes between a primary coder and a secondary coder for 20% of the data. After establishing reliability, the remaining dataset was coded by the primary coder. The primary coder was blinded to the goals/hypotheses of the study. As is evident from the average values, the healthy participants in this study showed some errors in IPS and motor coordination; however, there were little to no other experimenter errors. Later, we examined each of the trials including the erroneous trials to confirm if the profiles had any persistent motion artifacts or obvious outlier values compared to the other similar trials from each condition. Ultimately only 22.6% of overall data was eliminated due to persistent motion artifacts (19% of Watch, 24.3% of Do, and 22.4% of Together, ~6–7 of 8 trials/condition were used).

### Statistical analyses

To limit the number of comparisons and spurious results, we are conducting statistical analyses for average HbO_2_ data only. In addition, to avoid multiple, channel-wise comparisons, we also averaged data across channels within the same ROI based on our spatial registration output (see Figure 2C and Table 1 to see each of the 6 ROIs and constituent channels). All participants moved their right hand during the task, therefore, right hemisphere activation is considered ipsilateral, and left hemisphere activation is considered contralateral. Overall, we determined levels of activation for six ROIs including the contralateral/left and ipsilateral/right supero-anterior (SA), infero-posterior (IP), and infero-anterior (IA) regions (see Table 1). Using IBM SPSS, we conducted a repeated measures ANOVA with within-group factors of condition (Watch, Do, Together), hemisphere (left, right), and region of interest (SA, IP, IA) for average HbO_2_ values (SPSS, Inc.). To reconfirm our ROI results using representative channels, we have also conducted a repeated measures ANOVA using within-group factors of condition (Watch, Do, Together), hemisphere (left, right), and channel type (sensori-motor = 1 for left and 14 as right, IPL = 7 as left and 18 as right, STS = 12 as left and 23 as right as these channels best represented each of the 6 ROIs). The results of this second ANOVA are discussed within the Supplementary Materials. Greenhouse-Geisser corrections were applied when our data violated the sphericity assumption based on Mauchly's test. For multiple *post-hoc* comparisons, we have used the False Discovery Rate (FDR) method proposed by Singh and Dan (2006) for multichannel fNIRS data. We specifically used the Benjamin-Hochberg method wherein unadjusted *p*-values are rank ordered from low to high. Statistical significance is declared if the unadjusted *p*-value is less than *p*-value threshold. *p*-threshold was determined by multiplying 0.05 with the ratio of unadjusted *p*-value rank to the total number of comparisons (*p-threshold* for *i*^th^ comparison = 0.05 × *i*/*n*; where *n* = total number of comparisons).

## Results

A repeated measures ANOVA of condition × hemisphere × ROI revealed a main effect of condition [*F*_(2, 238)_ = 109.01, η^2^ = 0.47, *p* = 0.0001, Do and Together > Watch], hemisphere [*F*_(1, 119)_ = 24.9, η^2^ = 0.17, *p* = 0.0001, Left > Right], region [*F*_(2, 238)_ = 79.4, η^2^ = 0.40, *p* = 0.0001, IA and SA > IP], 2-way interactions between condition × hemisphere [*F*_(1.6, 194.1)_ = 23.3, η^2^ = 0.16, *p* = 0.0001], condition × region [*F*_(4, 476)_ = 7.3, η^2^ = 0.06, *p* = 0.0001], and hemisphere × region [*F*_(1.5, 183.1)_ = 3.5, η^2^ = 0.03, *p* = 0.04], as well as a 3-way condition × hemisphere × region interaction [*F*_(4, 476)_ = 2.4, η^2^ = 0.02, *p* = 0.04]. Next, we conducted simple *post-hoc* comparisons based on our aforementioned aims to examine the hemispheric, regional, and task-related differences in cortical activation.

### Hemispheric/regional differences

**Figure 5:**
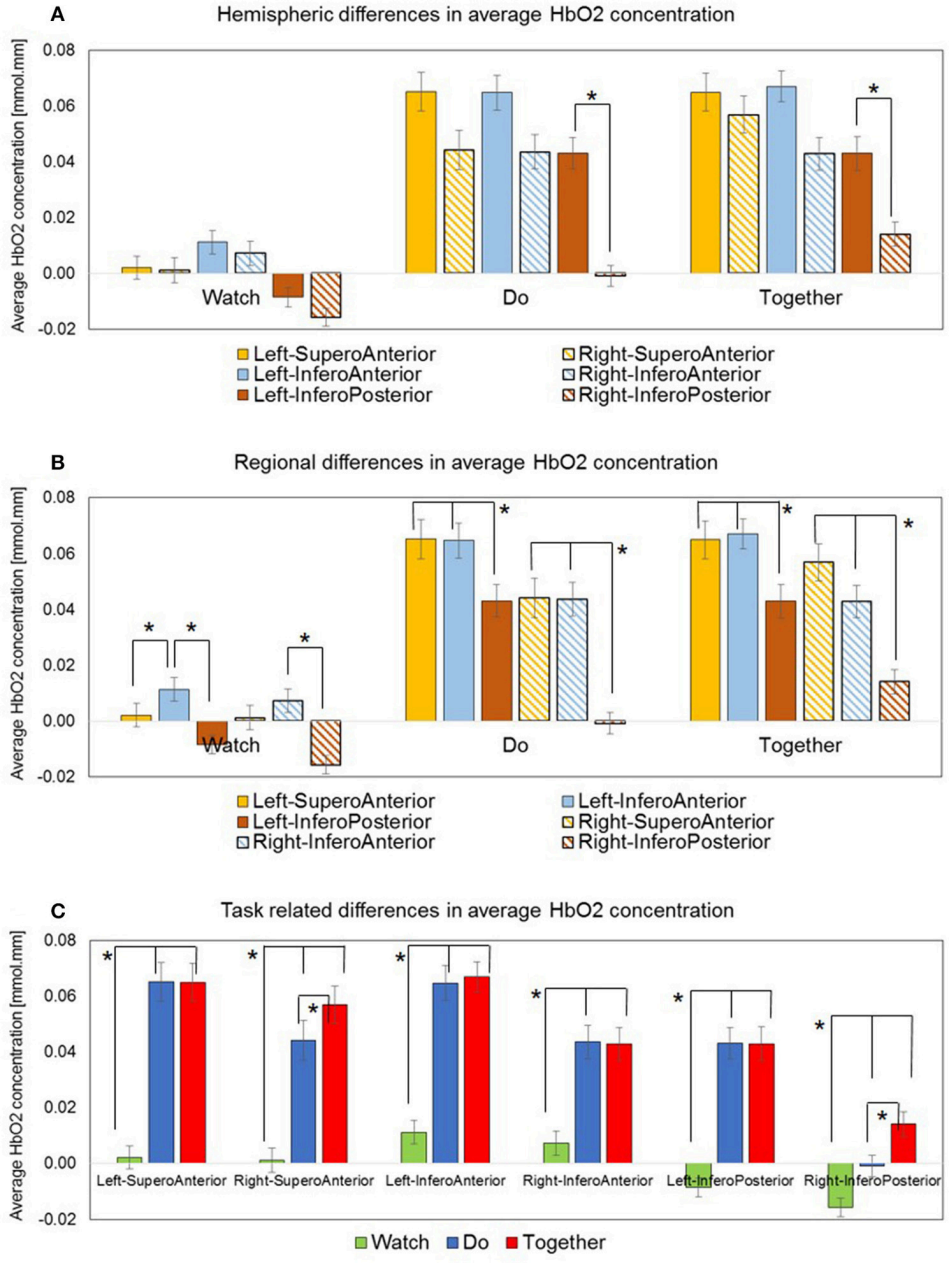
Average HbO_2_ concentration data is plotted in three ways: **(A)** Hemispheric differences: Only Left IP > Right IP. **(B)** Regional differences: Left IA > IP, Right IA > IP, Left IA > SA and a similar trend for Right IA > SA. **(C)** Task-related differences: Do and Together > Watch, for all ROIs. Together > Do for two ROIs, Right SA, and Right IP. ^*^Indicate significant differences.

During the Do and Together conditions, the contralateral IP region had significantly greater activation compared to the ipsilateral homologue (*p*-value for Do < 0.0001 and *p*-value for Together = 0.0002, see Figure 5A and Tables 2, 3). No other inter-hemispheric differences were significant. In terms of regional differences, during the Watch condition, bilateral IA regions had significantly greater activation compared to the other two regions (*p-*values between 0.03 and 0.0001, see the Watch bars in Figure 5B and Tables 2, 3). However, during the Do and Together conditions, there was significantly greater activation in the bilateral SA and IA regions compared to the bilateral IP regions (*p*-values between 0.0001 and 0.002, see the Do and Together bars in Figure 5B and Tables 2, 3).

**Table 2:** A listing of significant *p*-values from *post-hoc* testing.

**Comparison**	**Significant *p*-values**	**Direction of effect**
**MAIN EFFECTS**		
Condition	<0.0001	Do and Together > Watch
Hemisphere	<0.0001	Left > Right
Region	<0.0001	IA and SA > IP
**HEMISPHERIC DIFFERENCES (L VS. R)**		
Left IP vs. Right IP for Do	<0.0001	Left > Right
Left IP vs. Right IP for Together	0.0002	Left > Right
**REGIONAL DIFFERENCES**		
**Watch**		
Right, IA vs. IP	<0.0001	Right, IA > IP
Left, IA vs. IP	0.0001	Left, IA > IP
Left, IA vs. SA	0.03	Left, IA > SA
Right, IA vs. SA	0.06^∧^	Right, IA > SA
**Do**		
Left IA vs. IP	<0.0001	Right, IA > IP
Right, IA vs. IP	<0.0001	Left, IA > IP
Right, SA vs. IP	<0.0001	Left, SA > IP
Left, SA vs. IP	0.0003	Right, SA > IP
**Together**		
Right, IA vs. IP	<0.0001	Right, IA > IP
Left, IA vs. IP	<0.0001	Left, IA > IP
Right, SA vs. IP	<0.0001	Right, SA > IP
Left, SA vs. IP	0.002	Left, SA > IP
**CONDITION-RELATED DIFFERENCES**		
**Watch vs. Do**		
Left SA	<0.0001	Do > Watch
Left IA	<0.0001	Do > Watch
Left IP	<0.0001	Do > Watch
Right SA	<0.0001	Do > Watch
Right IA	<0.0001	Do > Watch
Right IP	0.0004	Do > Watch
**Watch vs. Together**		
Left SA	<0.0001	Together > Watch
Left IA	<0.0001	Together > Watch
Left IP	<0.0001	Together > Watch
Right SA	<0.0001	Together > Watch
Right IA	<0.0001	Together > Watch
Right IP	<0.0001	Together > Watch
**Do vs. Together**		
Right SA	0.0026	Together > Do
Right IP	0.006	Together > Do

∧ *Indicates a statistical trend*.

**Table 3:** Mean and standard error (SE) of activation based on HbO_2_ concentration values.

**Group activation data**	**Watch**		**Do**		**Together**	
	**Mean**	**SE**	**Mean**	**SE**	**Mean**	**SE**
**LEFT HEMISPHERE**						
Left SA/Left fronto-parietal	0.002	0.004	0.065	0.007	0.065	0.007
Left IA/Left superior temporal	0.011	0.004	0.065	0.006	0.067	0.005
Left IP/Left inferior parietal	−0.009	0.003	0.043	0.006	0.043	0.006
**RIGHT HEMISPHERE**						
Right SA/Right fronto-parietal	0.001	0.004	0.044	0.007	0.057	0.007
Right IA/Right superior temporal	0.007	0.004	0.044	0.006	0.043	0.006
Right IP/Right inferior parietal	−0.016	0.003	−0.001	0.004	0.014	0.004

### Task-related differences

**Figure 6:**
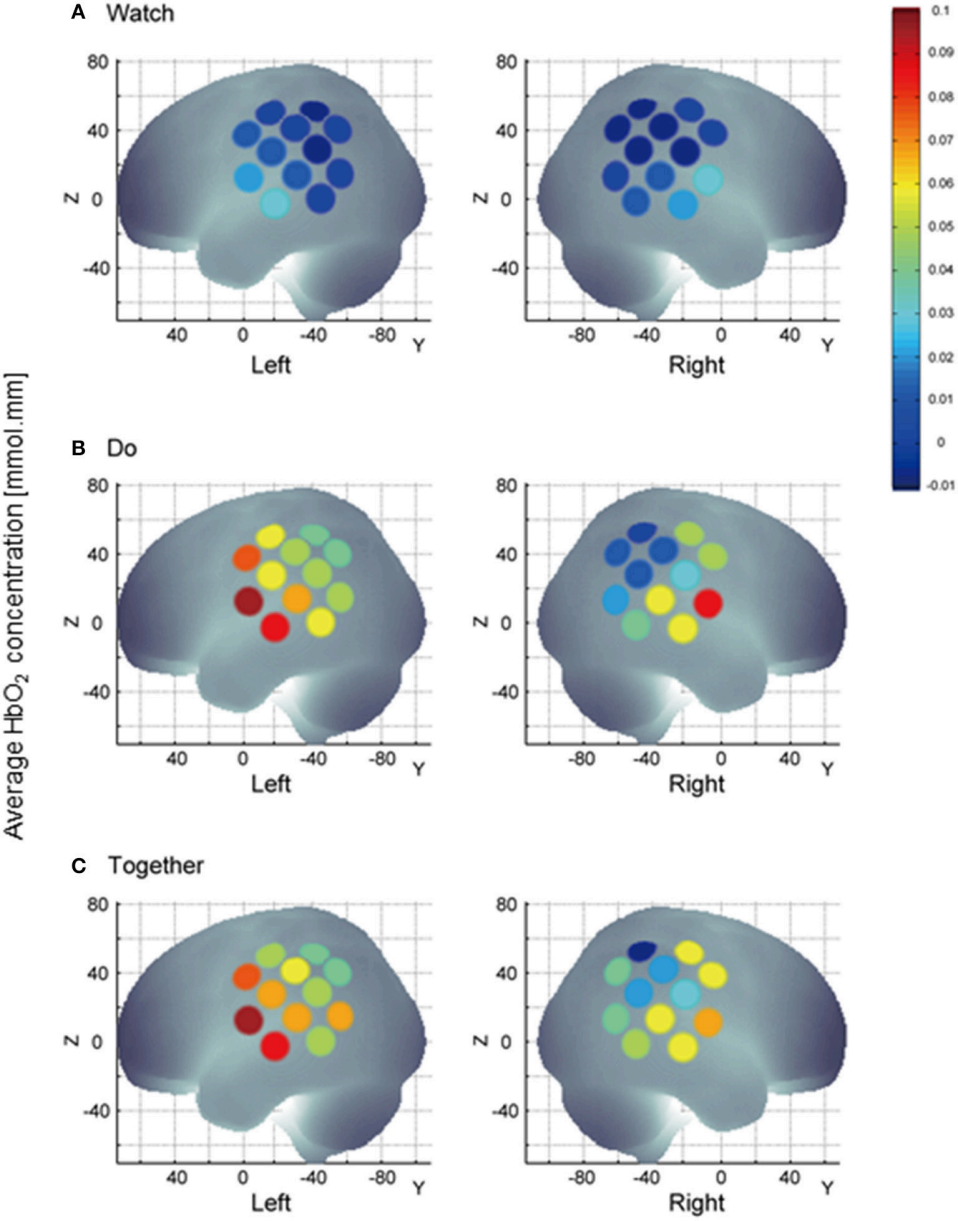
A visual representation of task-related channel activation; channel activation during the stimulation period is compared to its own baseline. HbO_2_ values on Y-axis range from 0 indicated by blue to 0.1 indicated by red and shades in between. During Watch condition **(A)**, channels 11, 23, and 24 representing the STS region are more active than other channels. Multichannel activation during the Do and Together condition vs. the Watch condition **(B,C)** vs. **(A)**. During the Together condition **(C)**, right channels 14, 15, 16, and 18 are more active vs. Do **(B)**.

Two out of the six ROIs, namely, ipsilateral or right SA and IP regions had significantly greater activation during the Together condition compared to the Do condition (*p*-value for right SA = 0.0026 and for right IP = 0.006, see Figure 5C, Do vs. Together and Tables 2, 3). The remaining four regions, namely, the ipsilateral IA and contralateral SA, IP, and IA had similar levels of activation during the Do and Together conditions. Lastly, for all ROIs, the Do and Together conditions had significantly greater activation compared to the Watch condition (*p*-values below 0.0001 for all six ROIs, see Figure 5C for Watch vs. Do and Watch vs. Together and Tables 2, 3). In addition, see Figure 6 for qualitative comparisons as well as additional Supplementary Materials for channel-specific differences in activation between the three conditions. Note that channel-specific comparisons yield results similar to that of the ROI-based comparisons.

## Discussion

The study of imitation/IPS control has been limited to hand motions and relatively unnatural fMRI environments. Few studies have assessed cortical activation patterns within imitation and sensori-motor networks during IPS tasks between two individuals during naturalistic fundamental movements such as reaching. In our study, we compared action observation, action execution, and IPS during a reach and cleanup task between pairs of healthy adults. Consistent with our original hypothesis, we found the following: In terms of hemispheric/regional differences, during the Do and Together conditions, the contralateral IP regions (inferior parietal regions covering IPL, SMG and AG) were more active than their ipsilateral homologue. During the Watch condition, bilateral IA regions (i.e., superior temporal regions covering STG and MTG) showed greater activation compared to other regions. During the Do and Together conditions, bilateral SA (i.e., fronto-parietal regions covering the precentral/post-central gyri and some portions of IFG) and IA regions (i.e., superior temporal regions covering STG and MTG) showed greater activation compared to the IP region (i.e., inferior parietal regions covering the IPL, SMG, and AG). In terms of task-related differences, the Do and Together conditions showed greater activation in the majority of the ROIs compared to the Watch condition. However, two out of the six ROIs showed greater activation during the Together condition compared to the Do condition, namely, the ipsilateral IP (i.e., inferior parietal regions covering the IPL, SMG, and AG) and ipsilateral SA regions (i.e., fronto-parietal regions covering the precentral/post-central gyri and some portions of IFG). It is important to note that for the remaining four regions, the Do and Together conditions had similar levels of activation.

### Greater inferior parietal cortex activation during action execution and IPS

During action execution and IPS, the left inferior parietal cortices showed more activation than their right-sided homologue. These findings fit with the notion that the left IPL (including the SMG, AG, and the intra-parietal sulcus) encodes kinematic aspects of the motor plan and perhaps the higher activation in the inferior parietal region during action execution and IPS may be linked to the planning requirements of the repetitive reach-grasp motions performed during the two conditions. Patients with left parietal lobe lesions produced several spatiotemporal errors during gesture imitation compared to the limited number of errors during gesture comprehension (Heilman and Gonzalez-Rothi, 1993; Halsband et al., 2001; Muhlau et al., 2005)). Furthermore, patients with left parietal lobe lesions produced significant errors during meaningless gestures compared to meaningful ones due to the greater difficulty in planning the kinematics of meaningless motor sequences (Goldenberg and Hagmann, 1997; Tessari et al., 2007). More recent work using transcranial magnetic stimulation to the anterior intraparietal sulcus and superior parietal lobule revealed their role in integrating target goals and developing an emerging action plan (Tunik et al., 2008). Similarly, a theta burst stimulation study during a human-avatar interaction task revealed that the anterior intra-parietal sulcus may encode shared goals of one's own and other's complementary actions (Sacheli et al., 2015). Taken together, multiple cortical regions within the left inferior parietal cortex may have contributed to the motor planning of the reach-grasp actions during the Do condition and/or the shared goals of the Together condition.

### Superior temporal cortex activation during action observation

During the action observation task, bilateral superior temporal cortices (i.e., STG and MTG) were most active compared to the SA and IP regions. These results fit with other fMRI findings of greater STS activation during action observation tasks (Montgomery et al., 2007; Molenberghs et al., 2010; Gatti et al., 2017). The STS region within the superior temporal cortex is considered important for processing and distinguishing social information such as biological motion, goal-directed actions of others, and mutual social gaze (Grossman and Blake, 2001; Pelphrey and Carter, 2008). Several fMRI studies have confirmed the role of STS in biological motion perception (Castelli et al., 2000; Grezes and Decety, 2001; Grossman and Blake, 2001; Pelphrey et al., 2003). Pelphrey et al. showed greater STS activation during observation of human or robotic motions compared to non-biological, object-related motions (Pelphrey et al., 2003). Hence, our finding of greater fNIRS-based activity in bilateral superior temporal cortices during the action observation condition is consistent with past fMRI studies. Furthermore, we extend the results of past fNIRS studies on social observation to a naturalistic, reach and cleanup task involving two individuals (Shibata et al., 2007; Shimada and Abe, 2009; Bolling et al., 2013). During a computerized ball toss game involving healthy adults, fNIRS-based activation was increased within the STS region when observing biological motion within a social inclusion context vs. a social exclusion context (Bolling et al., 2013). Therefore, our findings coincide with fMRI and fNIRS studies reporting bilateral STS activation during social observation of other's actions.

### Task-related similarities and differences in cortical activation—action execution influences IPS more than action observation

During the Do and Together conditions, bilateral SA and IA regions had greater levels of activation compared to the IP regions. SA region activation was not surprising because SA region comprised of pre- and post-central gyri or sensori-motor cortices (along with inferior frontal gyri) that are important for skilled motor performance (i.e., both Do and Together conditions required accurate reaching to targets such as blocks and the container) (Cincotta and Ziemann, 2008). Similarly, during the self-selected motor task (i.e., the Do condition), we found temporal cortex activation (i.e., STG and MTG) in spite of no overt social interactions between the participant and the tester. Note that testers were asked to avoid eye contact and overt social interactions with the participant during action execution. Additionally, we have viewed the video data to remove any Do trials that involved social interactions; however, the mere presence of the tester may have contributed to some of the STS activation. Our findings fit with the current fMRI literature in that two studies have reported significant activation in the STS region during action execution and imitation tasks (Montgomery et al., 2007; Molenberghs et al., 2010). During object-based gesture tasks, STS activation was greater bilaterally during action execution and action imitation compared to action observation (Montgomery et al., 2007). STS regions are said to provide a visual description of actions to the putative MNS (Iacoboni, 2005). Molenberghs et al. suggested that STS is not merely registering the biological motions during imitation but also encoding the visuomotor correspondence between one's own action and that of the partner. In fact, an fMRI study measuring cortical activation during observation of congruent vs. incongruent actions between two individuals revealed greater STS activation in the incongruent vs. congruent condition further corroborating the idea that STS may indeed be encoding visuomotor correspondences between individuals moving together (Shibata et al., 2011). STS region may be interacting with IPL to receive efference copies of the motor plans to match the performed actions with the visual descriptions of imagined or observed actions (Iacoboni, 2005; Montgomery et al., 2007).

In general, cortical activation during IPS was more similar to that of activation during action execution (not action observation). We believe that the challenges of imitation/IPS control stem from the complexity of motor components and not the observation component. It is often reported in the literature that simpler imitative tasks require less MNS activation compared to complex motor tasks and imitation performance is inextricably linked to its motor requirements such as body parts/joints involved as well as action complexity (Iacoboni, 2009; Gatti et al., 2017). To our knowledge, this is the first study to report greater fNIRS-based activation in the STS during action execution and imitation tasks compared to action observation. Only one other fNIRS study has reported greater activation in the STG during observation of appropriate socially cooperative actions between two individuals compared to inappropriate actions (Shimada and Abe, 2009).

While there were many similarities, there were only two clear differences in activation between the Do and Together conditions. Specifically, the right SA (pre- and post-central gyri and some IFG) and right IP (SMG and AG) regions were more active during the IPS condition compared to the action execution condition (see Figures 5C, 6, right sided activations). These findings also correspond with multiple past studies including a recent comprehensive meta-analysis on gestural imitation studies reporting greater bilateral activation in the frontal and inferior parietal cortices during imitation tasks compared to action execution and observation tasks (Aziz-Zadeh et al., 2006; Biermann-Ruben et al., 2008; Caspers et al., 2010). Lastly, both, Do and Together conditions led to greater activation in the majority of the ROIs compared to the Watch condition. This suggests that socially embedded actions such as IPS and imitation result in highest cortical demands followed by the execution condition, and lastly, the social observation/monitoring condition. During object-based gesture tasks and communicative gesture tasks, Montgomery et al. (2007) reported mostly similar activation between the action execution and imitation conditions but both movement conditions led to significantly greater activation in the IFG and IPL regions compared to the action observation condition. In terms of fNIRS literature, only two studies have reported greater IPL or IFG activity during simultaneous performance of cooperative actions with another partner (Egetemeir et al., 2011; Liu et al., 2015). During a table-setting task, Egetemeir et al. (2011) reported greater activation in bilateral IPL regions during a joint action condition compared to the solo action or observation condition. Similarly, when two adults engaged in a cooperation game, the fNIRS-based coherence patterns between their brain regions suggested that active following led to greater activation in the IFG (vs. passive following of a partner) (Liu et al., 2015). In short, both fMRI and fNIRS studies confirm that socially synchronous movements such as IPS and imitation require significant cortical activation beyond what is required in social observation or solo execution. Additionally, the apraxia literature offers further evidence on the role of IPL and IFG during object-based, goal-directed actions. Adults with parietal (IPL) and frontal (IFG) damage show significant impairments in gesture production, specifically, in reproducing imitated or instructed actions (Haaland et al., 2000; Goldenberg et al., 2007; Buxbaum et al., 2014). Both regions are considered important for goal directed actions; while IPL plans for the kinematic components of tool-based gestures, IFG is said to encode the postural components and goals of tool-based actions.

### Study limitations

This was our first study implementing various complex analytical methods for fNIRS data. For this reason, we did not take on whole brain assessment and limited our analysis to 24 data channels. In the future, we plan to use the full array of 52 channels of the ETG-4000 system to allow study of other related regions such as the motor, premotor, and prefrontal cortices. With our current analytical tools, we are unable to comment on the temporal patterns of activation, specifically, whether certain putative MNS regions activate before others. Second, in spite of the small number of channels, our multichannel fNIRS were affected by data loss; however, the proportion for data loss is consistent with other fNIRS studies. Third, we were unable to compare fNIRS patterns between the two individuals within each dyad. However, in the future, we plan to conduct brain coherence analyses between individuals during IPS and imitation tasks. For the movement tasks, especially, the IPS condition, we are unable to parse out the cortical effects of task complexity, working memory, vs. attention. Nevertheless, we controlled for attentional requirements by asking participants to focus on a cross hair during baseline periods. There was a small variation between trial lengths of each condition that may have affected our results; however, we have averaged activation data over the stimulation period to address this limitation. Our sample size is fairly small but is consistent with what has been used in past fMRI and fNIRS studies involving imitation/IPS tasks. Additionally, power analyses based on current data suggest that we have high statistical power for multiple data trends with a sample of 15 subjects. A well-known limitation of fNIRS is its inability to assess deeper brain structures. Last but not the least, variation in probe placement could have resulted in variability and inconsistency in spatial registration of data channels. However, we have made sure to place the cap in a consistent manner across all adults using the International 10–20 system and took pictures to ensure placement consistency.

### Clinical implications and future directions

Our study may have identified potential fNIRS-based neurobiomarkers associated with action observation, action execution, and IPS in fronto-parietal, inferior parietal, and superior temporal cortices. Our future studies will further investigate the validity of these findings by comparing similar tasks using fMRI and fNIRS. Currently, we are comparing IPS and imitation behaviors between individuals with and without ASD. Another study direction would be to examine fNIRS-based connectivity patterns between ROIs given reports of abnormal cortical connectivity in individuals with ASD. In the long-term, we would like to assess changes in cortical activation and connectivity following social-motor learning and therapeutic interventions offered to infants and children with ASD.

## Conclusions

The present study aimed to examine differences in activation patterns in the putative MNS regions during action observation, action execution, and IPS conditions of a fundamental reach and cleanup task in a group of healthy adults. We found that various putative MNS regions were active; specifically, the superior temporal cortices were active during action observation and the fronto-parietal and superior temporal cortices were more active during action execution and IPS. Furthermore, there was more bilateral activation in the fronto-parietal and inferior parietal regions during IPS compared to action execution and action observation conditions. Together, these findings highlight the importance of various cortical structures during IPS and imitation performance. Consistent with past studies on action imitation, socially synchronous movements involved more bilateral cortical activation. Lastly, we may have identified potential fNIRS-based neurobiomarkers for each component of IPS (action observation, execution, and both). These findings provide us a neuroimaging framework to study cortical impairments and to explore the value of fNIRS-based predictors to study effects of IPS-based interventions in children and adolescents with ASD.

## Author notes

- MH is currently a medical student at American University of Antigua, College of Medicine, Osbourn, Antigua and Barbuda.
- ST is currently a graduate student at Emory University Rollins School of Public Health, NE Atlanta, GA, United States.

## Author contributions

AB: Contributed through oversight and involvement in all aspects of this project including recruitment/screening, data collections, data analyses, and manuscript writing. MH, ST, and MC: Contributed to the project through help with recruitment, data collections and data analyses. JE and KP: Contributed to the project in the initial phases during task development and NIRS paradigm development. Specifically, JE wrote the E-Prime code used in this project. Finally, DT and his colleagues developed the anchor-based spatial registration approach to identify the cortical ROIs under each channel. DT contributed to the project by training the first author's lab in implementing his spatial registration, open-source MATLAB code. He also assisted in writing the spatial registration section and in preparing multiple figures for this manuscript.

### Conflict of interest statement

The authors declare that the research was conducted in the absence of any commercial or financial relationships that could be construed as a potential conflict of interest.
