# Supplementary material for: Cortical Activation during Action Observation, Action Execution, and Interpersonal Synchrony in Adults: A functional Near-Infrared Spectroscopy (fNIRS) Study
Source: Front Hum Neurosci. 2017 Sep 5;11:431. doi: 10.3389/fnhum.2017.00431 (PMC5591977; doi:10.3389/fnhum.2017.00431)
Supplement: Supplementary file 1 [file DataSheet1.pdf]

Supplementary Material

We have conducted another repeated-measures ANOVA to determine channel-based differences. We relied on single channel with maximum coverage over each ROI (SA, IA, and IP). Selected channels were 70-100% over specific ROIs. Specifically, channels 3 and 17 represented left and right SA or Pre/post CG, channels 7 and 18 represented left and right IPL regions, 12 and 23 represented left and right STS regions (see Table 1 and Figure 2).

A repeated measures ANOVA of condition x hemisphere x channel type revealed a main effect of condition ($F(2, 238)=75.2$, p=0.0001), hemisphere ($F(1, 119)=5.8$, p=0.01), channel type ($F(2, 238)= 22.3$, p=0.0001), 2-way interactions between condition x hemisphere ($F(1.6, 194.2)= 13.3$, p=0.0001), and condition x channel type ($F(3.6, 434.1)=3.1$, p=0.02), and a hemisphere x channel type ($F(1.8, 210.5) = 6.9$, p=0.002), and a 3-way condition x hemisphere x channel interaction ($F(3.1, 372.2) = 2.5$, p=0.04). Note that the results of this ANOVA are similar to the one performed in the main body of the paper wherein we used region-wise data or an average of activations of those channels that overlay a particular ROI.

In terms of hemispheric differences, we found greater left IP activation (channel 7) compared to its ipsilateral homologue (channel 18) for the Do (p=0.0007) and Together (p=0.0001) conditions only (Figure 6B and 6C). In terms of regional differences, for the Watch condition we found greater bilateral IA activation (i.e., channels 12 and 23 > remaining channels, *p*-values ranged from 0.0001 to 0.0002, see Figure 6A) compared to other regions. During the Do and Together conditions we found greater activation in the SA and IA region's representative channels compared to that of the IP region (i.e., channels 14 and 23 > channel 18, *p*-values < 0.0001). In terms of task-related differences, right SA (channel 14) and right IP (channel 15) regions had greater activation during the Together condition compared to the Do condition (*p*-values between

0.0009 and 0.0013, see Figure 6B and 6C, Do vs. Together). Similar statistical trends were noted for channels 16 (p-value=0.032) and 18 (p-value=0.037) but they did not remain significant after applying the FDR thresholds. Lastly, for all representative channels, the Do and Together conditions had greater activation compared to the Watch condition (*p*-values between 0.0001 and 0.006 for representative channels, see Figure 5C and Figure 6A, 6B, and 6C Watch vs. Do & Watch vs. Together). Note that these patterns of channel-based differences in activation are similar to the patterns observed in the region-based analyses discussed within the main body of this manuscript.
